# High-performance superconducting quantum processors via laser annealing of transmon qubits

**DOI:** 10.1126/sciadv.abi6690

**Published:** 2022-05-13

**Authors:** Eric J. Zhang, Srikanth Srinivasan, Neereja Sundaresan, Daniela F. Bogorin, Yves Martin, Jared B. Hertzberg, John Timmerwilke, Emily J. Pritchett, Jeng-Bang Yau, Cindy Wang, William Landers, Eric P. Lewandowski, Adinath Narasgond, Sami Rosenblatt, George A. Keefe, Isaac Lauer, Mary Beth Rothwell, Douglas T. McClure, Oliver E. Dial, Jason S. Orcutt, Markus Brink, Jerry M. Chow

**Affiliations:** IBM Quantum, IBM T. J. Watson Research Center, Yorktown Heights, NY 10598, USA.

## Abstract

Scaling the number of qubits while maintaining high-fidelity quantum gates remains a key challenge for quantum computing. Presently, superconducting quantum processors with >50 qubits are actively available. For these systems, fixed-frequency transmons are attractive because of their long coherence and noise immunity. However, scaling fixed-frequency architectures proves challenging because of precise relative frequency requirements. Here, we use laser annealing to selectively tune transmon qubits into desired frequency patterns. Statistics over hundreds of annealed qubits demonstrate an empirical tuning precision of 18.5 MHz, with no measurable impact on qubit coherence. We quantify gate error statistics on a tuned 65-qubit processor, with median two-qubit gate fidelity of 98.7%. Baseline tuning statistics yield a frequency-equivalent resistance precision of 4.7 MHz, sufficient for high-yield scaling beyond 10^3^ qubit levels. Moving forward, we anticipate selective laser annealing to play a central role in scaling fixed-frequency architectures.

## Teaser

Laser-based thermal annealing is demonstrated for high-yield scaling of high-performance superconducting quantum processors.

## INTRODUCTION

Recent technological advances have enabled rapid scaling of both the physical number of qubits and computational capabilities of quantum computers *(**1*–*5**)*. The distinction between classical and quantum computing arises from the exponentially larger computational subspace available to qubits (quantum bits), which may be set to nonclassical superpositions and entangled states. Existing multiqubit systems built on superconducting circuit quantum electrodynamics (cQED) architectures (*6*, *7*) have been used in applications ranging from early implementations of Shor’s factoring algorithm (*8*) to quantum chemistry simulations (*9*–*11*) and accelerated feature mapping in machine learning *(**12*–*14**)*. Presently, solid-state cQED-based processors have substantially increased in scale (*2*, *15*), with dozens of physical qubits demonstrated on a single quantum chip. As gate fidelities improve and eventually reach thresholds required for fault tolerance, quantum advantage will be exploited to simulate complex molecular dynamics and implement quantum algorithms on practical scales (*9*, *16*–*18*). To track the continual progression of quantum processing power, the quantum volume (QV) metric is used as an overall measure of the computational space available for a given processor (*5*).

Among the major classes of superconducting qubits, fixed-frequency transmons (*3*, *7*) operating in the *E_J_ >> E_C_* regime are attractive for their low charge dispersion, yielding relatively noise-immune qubits with coherence times (*T*_1_, *T*_2_) exceeding 100 μs (*19*). Transmon qubits are amenable to high-fidelity (>99.9%) single-qubit gate operations (*3*), and two-qubit entangling cross-resonance (CR) gates (*20*) are realized via static qubit-qubit coupling activated by an all-microwave drive scheme (*3*, *21*). A key requirement to enable high-fidelity CR gates involves the selective addressability of fixed-frequency transmons, as well as precisely engineering their computational ∣0〉 → ∣1〉 transitions (*f*_01_) for optimal two-qubit interaction (*3*). For example, suboptimal *f*_01_ separation between neighboring qubits reduces ZX coupling strength, while higher-order static ZZ interactions cause accumulation of two-qubit errors and “spectator” error propagation across the lattice (*22*). The most likely frequency collisions and corresponding tolerance bounds resulting from frequency crowding of lattice transmons have been quantitatively enumerated in (*23*).

The principal challenge for scaling fixed-frequency architectures is mitigating errors arising from lattice frequency collisions. Typical fabrication tolerances for transmon frequencies range from 1 to 2%, with uncertainties dominated by the 2 to 4% variation in tunnel junction resistance *R_n_* (*23*, *24*). Thermal annealing methods to adjust and stabilize post-fabrication *R_n_* (and correspondingly, transmon frequencies *f*_01_) have been explored previously in (*25*–*28*). More recently, the LASIQ (Laser Annealing of Stochastically Impaired Qubits) technique was introduced to increase collision-free yield of transmon lattices by selectively trimming (i.e., tuning) individual qubit frequencies via laser thermal annealing (*23*). The LASIQ process sets *R_n_* with high precision, and *f*_01_ could be predicted from *R_n_* according to a power-law relationship resulting from the Ambegaokar-Baratoff relations and transmon theory (*7*, *29*). An empirical *f*_01_(*R_n_*) scatter of σ*_f_* ≃ 14 MHz limited the ability to predict *f*_01_ from *R_n_*, resulting in a post-tune frequency precision of equivalently ~14 MHz (*23*).

Here, we demonstrate LASIQ as a scalable process tool used to reduce two-qubit gate errors by systematically trimming lattice transmon frequencies to desired patterns. We show laser tuning results on statistical aggregates of >300 tuned qubits and demonstrate LASIQ baseline frequency-equivalent resistance tuning precision of 4.7 MHz from empirical *f*_01_(*R_n_*) correlations, reaching this precision with 89.5% success rate. On the basis of cryogenic *f*_01_ measurements from our laser-tuned processors, we empirically find a frequency assignment precision of 18.5 MHz, which, in addition to the LASIQ tuning precision, includes all deviations arising from pre-cooldown steps including post-tuned chip cleaning and bonding processes. Our results indicate that the precision of trimming *f*_01_ is dominated by the residual *f*_01_(*R_n_*) scatter of untuned qubits (σ*_f_* = 18.1 MHz) and is not limited by the LASIQ trimming process itself.

In addition to scaling the number of tuned qubits, we measure functional parameters of multiqubit chips (coherence and two-qubit gate fidelity) to ensure high processor performance. We assess the impact of LASIQ tuning on qubit coherence using a collection of composite (partially tuned) processors, showing aggregate mean *T*_1_ and *T*_2_ times of 79 ± 16 μs and 69 ± 26 μs, respectively, with no statistically significant variation between the tuned and untuned groups. Our LASIQ process has been broadly used for precise frequency control of post-fabricated 27-qubit Falcon processors, including the recent QV-128 *ibmq_montreal* system (*5*, *30*). We demonstrate LASIQ scaling capabilities by tuning a 65-qubit Hummingbird processor (cloud-accessible as *ibmq_manhattan*), with a median two-qubit gate fidelity of 98.7% based on randomized benchmarking (*3*, *31*). As a scalable frequency trimming tool for fixed-frequency transmon architectures, we envision the LASIQ process to be widely implemented in future generations of superconducting quantum systems.

## RESULTS

### Tuning a 27-qubit Falcon processor

**Fig. 1.**
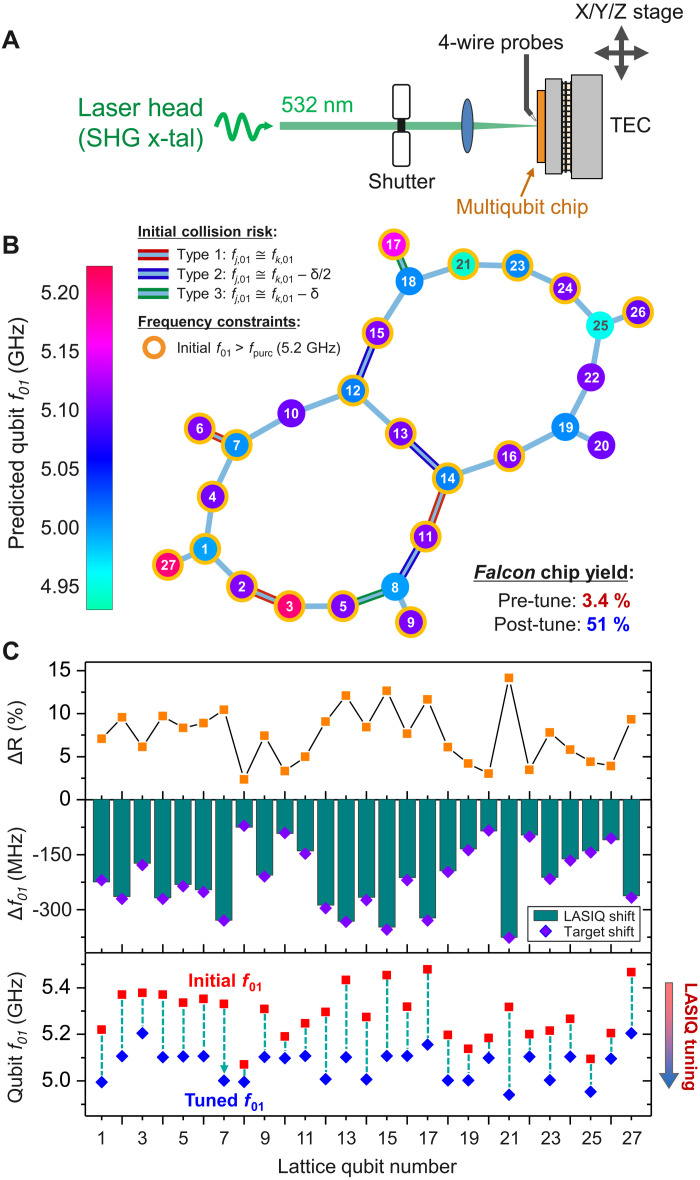
Example of a LASIQ anneal process. (**A**) Outline of the laser trimming setup (*23*). A 532-nm second-harmonic generation laser is sequentially focused on the junctions of a multiqubit quantum processor, with thermal annealing to selectively decrease qubit frequencies (*f*_01_) for collision avoidance. (**B**) Example of a tuned 27-qubit Falcon lattice. Final predicted *f*_01_ are depicted as a heatmap, with initial high-risk NN collision pairs highlighted, and orange outlines indicating initial *f*_01_ above the bandwidth of Purcell protection. After LASIQ, collision and frequency constraints are resolved. (**C**) Detail of qubit anneals. The bottom panel indicates the initial (red) and final (blue) predicted *f*_01_ showing the qubits tuned to distinct frequency set points. The middle panel indicates the tuning distance (monotonic negative shifts), along with the desired target shifts (purple diamonds), with an RMS deviation (i.e., frequency-equivalent resistance tuning precision) of 4.8 MHz, as determined from empirical *f*_01_(*R_n_*) correlations. The top panel depicts the corresponding junction resistance shifts, achieving tuning ranges up to 14.2%.

As a practical demonstration of frequency trimming, a 27-qubit Falcon processor is tuned to predicted frequency targets using the LASIQ setup shown in Fig. 1A (described in Materials and Methods). The Falcon chip series are based on a heavy-hexagonal lattice, which contains the distance-3 hybrid surface and Bacon-Shor code for error correction (*32*). For this demonstration, all measurements were performed at ambient conditions, with resulting tuned frequencies estimated from empirical *f*_01_(*R_n_*) correlations. The tuned lattice is depicted in Fig. 1B, with a color heatmap indicating the post-LASIQ frequency predictions. Nearest-neighbor (NN) qubit frequency spacing (Δ*f*_NN_) can be visually seen to be separated by 50 MHz ≤ |Δ*f*_NN_| ≤ 250 MHz, placing qubits comfortably within the straddling regime for high-ZX interaction (*33*). NN collisions have been avoided with twice the collision tolerance (2Δ_c_, see the Supplementary Materials) from bounds described in (*23*) to protect against two-qubit state hybridization and improve chip yield. Before tuning, high-risk pairs within 2Δ_c_ collision bounds have edge borders highlighted as indicated in the lattice graph and are resolved after the LASIQ process is complete. On the basis of Monte Carlo models with a conservative post-tune spread estimate of σ*_f_* = 20 MHz (see the next section), we demonstrate substantial increase in the yield rate from 3.4 to 51% for achieving zero NN collisions, corresponding to 15× yield improvement after LASIQ tuning (see the Supplementary Materials). The target qubit *f*_01_ patterns corresponding to Fig. 1B are shown in Fig. 1C (bottom), where initial *f*_01_ (red) are progressively tuned until they reach predefined and distinct frequency set points (blue).

**Fig. 2.**
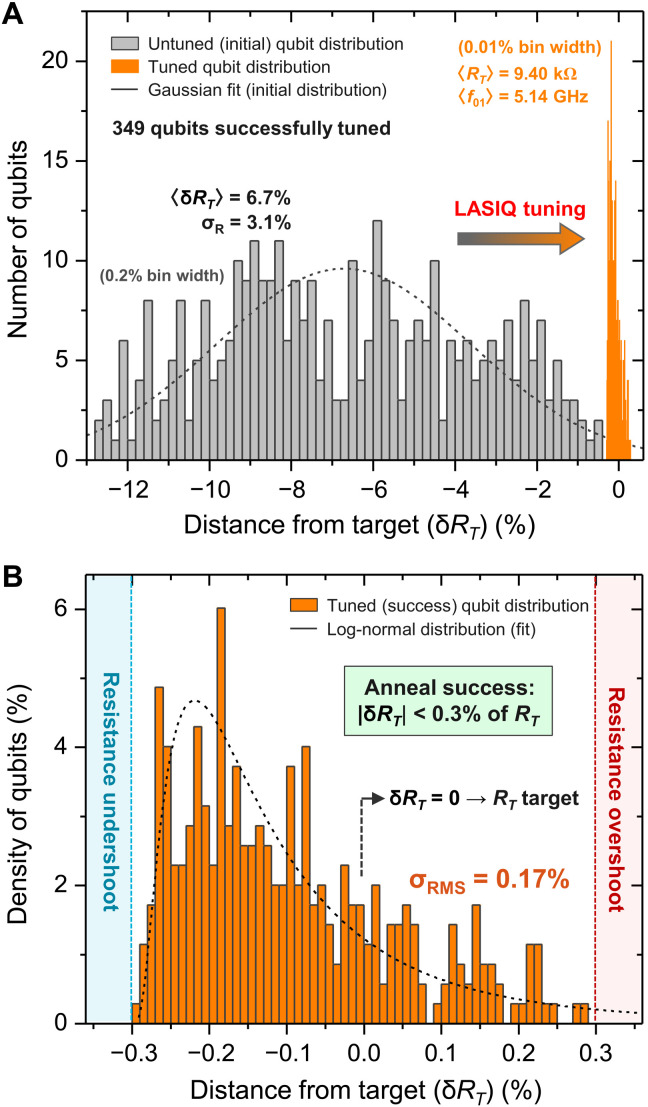
LASIQ tuning outcome statistics. (**A**) Initial distribution (gray) of qubits that were successfully tuned to target (orange). The distance from target δ*R_T_* is the tuning differential normalized to the final target resistance *R_T_*. Orange bars indicate the final distribution (20× reduced bin width for clarity) and show the 349 qubits tuned to success. (**B**) Expanded view of the orange distribution shown in (A). Anneal success is defined as a tuned resistance within 0.3% of *R_T_*, which was reached by all displayed qubits, and 89.5% of the 390 tuned qubits (details in the Supplementary Materials). The blue/red regions indicate undershoot/overshoot, respectively. A log-normal fit is shown by the black curve, which supports the interpretation of LASIQ tuning as an incremental resistance growth process.

In addition to NN collision avoidance, all qubits have been tuned to targets at or below *f*_purc_ = 5.2 GHz, which, in our Falcon design, is the cutoff for maintaining good Purcell protection and avoiding radiative qubit relaxation (*34*). Because of the monotonic increase of junction resistance (*R_n_*) intrinsic to the laser anneal process, qubit frequencies are “trimmed” (i.e., reduced) to desired *f*_01_ values. The LASIQ process is engineered to proceed until *R_n_* for all junction reside within 0.3% of the target *R_T_* [corresponding to ~10 MHz for typical *f*_01_(*R_n_*) correlation coefficients], although a nominal LASIQ approach will typically outperform this upper precision bound (see the next section). The middle panel of Fig. 1C shows desired target shifts (purple diamonds) superimposed on final predicted frequency tuning amplitudes (green bars), indicating excellent agreement with desired target values. The root mean square (RMS) *R_n_* deviation from *R_T_* for this processor is 0.16%, corresponding to a frequency-equivalent resistance tuning precision of 4.8 MHz [using nominal *f*_01_(*R_n_*) power-law coefficients for this sample] and is consistent with LASIQ baseline tuning precision as described in Fig. 2. As seen from the middle panel, target *f*_01_ shifts range from 75 MHz (qubit 8) to 375 MHz (qubit 21) in Fig. 1C, corresponding to resistance shifts up to Δ*R_n_* = 14.2%. On the basis of separate calibration measurements over standard junction arrays, resistance tuning peaks at ~14%, and therefore, tuning plans for each processor are designed to be constrained within attainable targets. Upon completion of the LASIQ process, a typical quantum processor is cooled and screened for coherence and single/two-qubit gate fidelity, and assessments of QV are performed (*5*). Two-qubit gate error statistics of a tuned and operational 65-qubit Hummingbird processor are shown in the “Qubit coherence and gate fidelity” section.

### LASIQ tuning precision

In prior work, tuning precision estimates were limited by the imprecision in predicting *f*_01_ from room temperature *R_n_* measurements (*23*). Thus, only an upper bound of tuning precision could be determined (σ*_f_* ≃ 14 MHz). Presently, we address the limits of LASIQ tuning precision as limited by the process itself, rather than our ability to predict cryogenic *f*_01_(*R_n_*). We analyze a large sample of 390 tuned qubits, 349 of which successfully tuned to target (defined in the previous section as achieving junction resistance within 0.3% of *R_T_*), yielding an aggregate tuning success rate of 89.5% for this experiment. The initial and final distributions of successfully tuned qubits are shown in Fig. 2. We note here that a full range of tuning (Δ*R* up to ~14% with respect to initial junction *R_n_*) was performed for this experiment, with anticipated failure rates being weighted toward tuning extremes (Δ*R* > 14% increases undershoot risk, while low tuning Δ*R* < 1% results in increased overshoot risk). These extremes are readily avoided during *f*_01_ tuning plan assignment. A detailed analysis of tuning statistics and success rates has been performed for all qubits (see the Supplementary Materials).

**Fig. 3.**
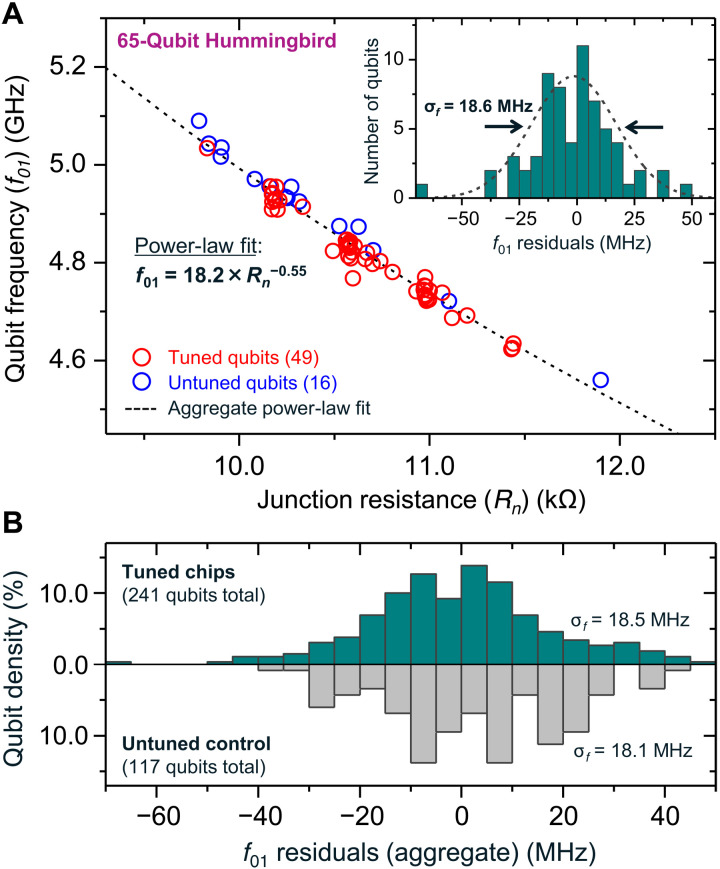
Frequency assignment precision based on statistical aggregates of tuned 27-qubit Falcon and 65-qubit Hummingbird processors. (**A**) Resistance (*R_n_*) to frequency (*f*_01_) correlation for a tuned Hummingbird processor. Cryogenic *f*_01_ measurements are plotted against measured junction resistances *R_n_*, with a power-law curve superimposed on the measured data. Both tuned (49 qubits) and untuned (*16*) qubits are depicted. The inset shows a histogram of residuals with an SD of 18.6 MHz, indicating the practical precision to which we may assign qubit frequencies. (**B**) The top panel shows statistical precision analysis performed for a total of 241 tuned qubits from a combination of Falcon and Hummingbird chips, with aggregate *f*_01_ residuals from individual power-law regressions for each chip. The bottom panel shows identical analysis performed for 117 untuned qubits from both processor families. Cryogenic *f*_01_ measurements yield 18.5- and 18.1-MHz spread for tuned and untuned qubits, respectively, indicating that the LASIQ process does not significantly affect the overall spread of qubit frequencies before preparatory chip cleaning, bonding, and cooldown processes.

To understand the consequences of the incremental approach to *R_T_*, aggregate tuning statistics of the 349 successfully tuned qubits are depicted in Fig. 2A. *R_n_* of the initial qubits (gray histogram) are tuned to a tight tolerance about the targets (orange), as displayed on an *R_T_* normalized scale. A Gaussian fit approximates the initial resistance distribution, yielding a mean fractional tuning distance of 6.7% (with respect to *R_T_*). The final distribution is magnified in Fig. 2B with a superimposed log-normal curve fit, a characteristic distribution for fractional growth processes consistent with incremental anneals during the LASIQ process. Averaging over the entire distribution yields an RMS error deviation σ*_R_* = 0.17% from *R_T_*, corresponding to σ*_f_* = ∂*f*_01_/∂*R_n_* ∙ σ*_R_* = 4.7 MHz as determined by empirical *f*_01_(*R_n_*) correlations from our 65-qubit Hummingbird processor (Fig. 3). Our determination of LASIQ resistance precision shows that the fundamental tuning performance is well below the upper bound of ~14 MHz determined in (*23*), where it was also noted that this bound was dominated by residual scatter in the prediction of cryogenic *f*_01_ from room temperature *R_n_*. On the basis of Monte Carlo simulations, a precision of ≤6 MHz is required for high-yield scaling for quantum processors beyond 10^3^ qubits (*23*). Therefore, our baseline frequency-equivalent resistance precision of σ*_f_* < 5 MHz demonstrates LASIQ as a viable post-fabrication trimming process for high-yield scaling of fixed-frequency transmon processors.

In addition to the residual *f*_01_(*R_n_*) prediction scatter, a number of preparatory cleaning, bonding, and processor mounting steps are undertaken for our quantum processors in the pre-cooldown stage. A natural question therefore arises as to the practical precision achieved for frequency assignment with the inclusion of these processes. Figure 3A shows cryogenic *f*_01_ plotted against post-LASIQ measurements of *R_n_* on a 65-qubit Hummingbird processor, which comprised 49 tuned qubits and 16 untuned qubits. After tuning, the processor underwent plasma cleaning and flip-chip bump-bonding to an interposer layer before mounting in a dilution refrigerator for cooldown and screening (see Materials and Methods). The entire process occurred within a 24-hour span to minimize the impact of aging and drift on the junction resistances (and therefore qubit frequencies). A power-law fit (dashed curve) conforms well to both the tuned and untuned qubits, indicating that no appreciable relative shifts due to LASIQ tuning have occurred, and that the same *f*_01_(*R_n_*) prediction may be adequately used in both cases. We note the slight deviation of the power-law fit exponent (−0.55) from the nominal one-half expected from the Ambegaokar-Baratoff relations and transmon theory (*7*, *29*), which is attributed to nonidealities in predicting *f*_01_ from room temperature *R_n_*. Nevertheless, this deviation to first order does not affect the relative frequency spacing between qubits and, therefore, does not affect the precision to which we assign qubit frequency spacing for collision avoidance. The effective *f*_01_ assignment precision may be determined by the residuals of the power-law fit as shown in Fig. 3A (inset), outlined by a Gaussian distribution with σ*_f_* = 18.6 MHz spread, corresponding to 0.38% of the mean qubit frequency (4.84 GHz), and defines the practical *f*_01_ assignment precision for this chip.

Figure 3B shows a similar analysis performed over a statistical sampling of 241 qubits from seven Falcon- and two Hummingbird-tuned processors. Each processor sample was fit to an individual *f*_01_(*R_n_*) curve, and residuals are aggregated in the upper histogram (dark green), with 1-σ*_f_* spread of 18.5 MHz, consistent with the single-processor sample observed in Fig. 3A. Results from control (untuned) qubits are shown in the bottom panel histogram (gray), which are *f*_01_(*R_n_*) residuals extracted from 117 untuned qubits on composite (partially tuned) processors, yielding 1-σ*_f_* spread of 18.1 MHz. We may therefore conclude that the *f*_01_ assignment imprecision resulting from our LASIQ process is a negligible contributor to the overall frequency spread of the qubits. We note that although our residual value for both tuned and untuned samples is larger than the ~14 MHz demonstrated in (*23*), our Falcon and Hummingbird processors undergo a greater number of preparatory steps before cooldown, and a certain amount of deviation is anticipated. The prediction imprecision of *f*_01_(*R_n_*) remains the dominant contributor to overall spread, with relatively smaller contributions from post-tune drifts and pre-cooldown processes. Therefore, room remains for improving frequency predictions to reach single-MHz levels dictated by the ~5 MHz LASIQ baseline frequency-equivalent tuning precision as shown in Fig. 2B (details in Discussion).

### Qubit coherence and gate fidelity

**Fig. 4.**
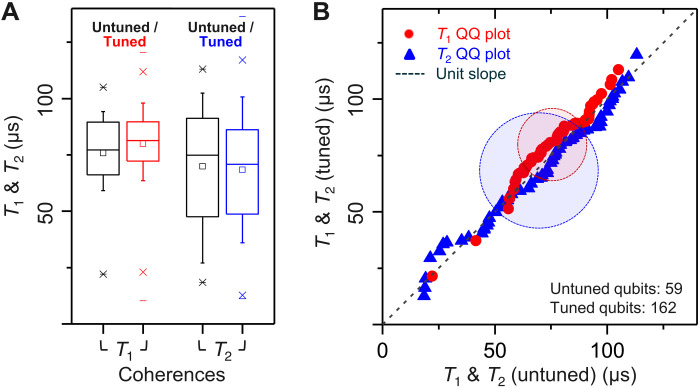
Impact of LASIQ tuning on qubit relaxation (*T*_1_, red) and dephasing (*T*_2_, blue), using composite (partially tuned) Hummingbird processors. Qubit coherences on four Hummingbird chips are analyzed. On each chip, both untuned and tuned qubits were simultaneously measured, for a total statistical sample of 59 untuned and 162 tuned qubits. (**A**) Box plots of *T*_1_ and *T*_2_ distributions (with interquartile box range, 10 to 90% whiskers, 1 to 99% outliers indicated by crosses and minima/maxima by horizontal markers). Coherence distributions show no statistically significant difference in untuned as compared with LASIQ-tuned qubit populations. (**B**) Illustrates this comparison as a quantile-quantile (QQ) plot of the *T*_1_ and *T*_2_ distributions. Each point represents a comparison between estimated quantiles from the set of 59 untuned qubits against the interpolated quantiles of the 162 tuned qubits. Good linearity with respect to unit slope indicates a close match of the coherence distributions in tuned and untuned qubit populations. Mean values agree robustly within statistical error bounds. For tuned (untuned) qubits, 〈*T*_1_〉 = 80 ± 16 μs (76 ± 15 μs) and 〈*T*_2_〉 = 68 ± 25 μs (70 ± 26 μs). The shaded ovals are centered on the mean coherence times and have 1-σ extent in relaxation and dephasing times.

Maintaining high qubit coherence is an essential component of high-fidelity single- and two-qubit gates, in addition to precise frequency control. To empirically determine the impact of laser tuning on qubit coherence, a composite (partially tuned) set of four Hummingbird processors were cooled and coherence was assessed. The composite chips allow direct comparison of tuned and untuned qubits drawn from the same initial population. Of a total 221 measured qubits from the four composite processors, 162 were LASIQ-tuned and 59 were left untuned (73% fractional tuning rate). The statistical aggregates of tuned and untuned (*T*_1_, red) and dephasing (*T*_2_, blue) times are shown in Fig. 4. For tuned (untuned) qubits, 〈*T*_1_〉 = 80 ± 16 μs (76 ± 15 μs) and 〈*T*_2_〉 = 68 ± 25 μs (70 ± 26 μs). Aggregate (including both tuned and untuned qubits) relaxation and dephasing times are 79 ± 16 μs and 69 ± 26 μs, respectively. Box plots (interquartile box range, with 10 to 90% whiskers) shown in Fig. 4A demonstrate that, within statistical error, the LASIQ process introduces no variation in qubit coherence. Figure 4B offers a detailed comparison of the tuned and untuned coherence distributions on a quantile-quantile plot. Good correspondence is observed with respect to linear unity slope, indicating close agreement between the distributions. Visual indicators of the mean 〈*T*_1_〉 and 〈*T*_2_〉 and 1-σ bounds are shown in the shaded ovals. Together, these comparisons of statistical distributions demonstrate the negligible effect of the LASIQ process on qubit coherence.

**Fig. 5.**
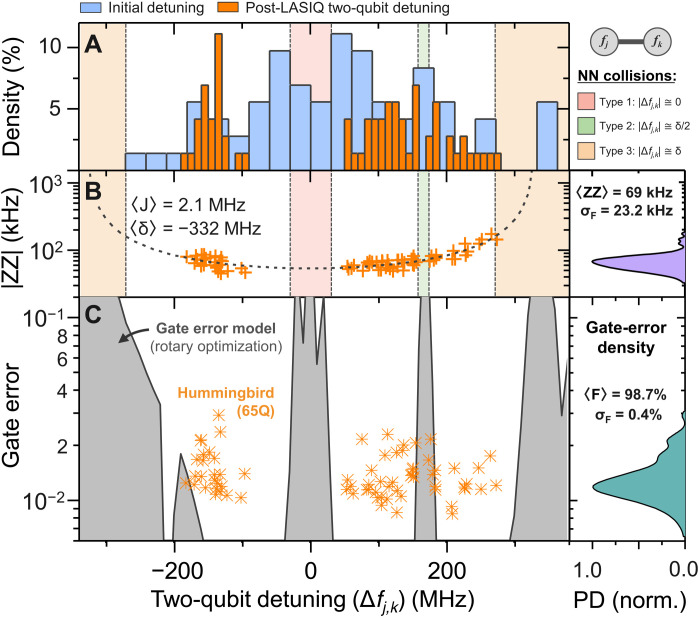
Gate errors of a 65-qubit Hummingbird processor after LASIQ tuning. (**A**) Distribution of tuned two-qubit *f*_01_ separation (orange), along with the initial (pre-LASIQ) distribution (blue), indicating high density of collisions and gate errors before LASIQ tuning. (**B**) Achieved ZZ distribution after LASIQ tuning, indicating well-tailored separation near null-detuning (type 1 NN collision), while maintaining a tight ZZ spread with 69-kHz median. A kernel density estimator (KDE) is used to calculate the ZZ probability density (right). (**C**) Measured CNOT (Controlled NOT) gate errors as a function of two-qubit detuning (orange points), yielding a median gate fidelity of 98.7% for the LASIQ-tuned Hummingbird (the corresponding KDE distribution of gate errors is shown on the right panel). The shaded (gray) regions indicate approximative error-rate projections based on CR gate error modeling (*35*), incorporating typical qubit interaction parameters (frequency and anharmonicity, qubit coupling, and gate times), with optional rotary echo pulsing for error minimization.

As a practical demonstration of LASIQ tuning capabilities, a 65-qubit Hummingbird processor is laser-tuned and operationally cloud-accessible as *ibmq_manhattan*. In a similar fashion to that shown in Fig. 1B, the LASIQ tuning plan is generated by ensuring avoidance of NN level degeneracies while maintaining level separation within the straddling regime *(**33**)*. After LASIQ, the 65-qubit processor was cooled and qubit frequencies were measured, with density of frequency detuning between two-qubit gate pairs shown in Fig. 5A (orange, 10 MHz bin width), along with the initial two-qubit detuning (blue, 30 MHz bins). As tuned, our processor consists of 72 operational two-qubit CR gates, corresponding to a 100% yield of working two-qubit gates, with gate durations ranging from 250 to 600 ns. Collision boundaries [2Δ_c_ bounds as derived from (*23*, *33*); see the Supplementary Materials] for NN degeneracies are shown in the background, consistent with those used in Fig. 1A, indicating that ~20% of gates are within “high-risk” collision zones. Monte Carlo yield modeling of the untuned (as-fabricated) 65-qubit Hummingbird chip indicates an average of 12 NN collisions (assuming nominal 20 MHz spread with Δ_c_ collision bounds), with an effectively null yield of zero NN collisions. These yield and collision outcomes are typical of untuned processor samples of this size. After LASIQ, the collisions are resolved, as evidenced by the high median two-qubit gate fidelity (98.7%) shown in the lower panel. The corresponding ZZ statistics are shown in Fig. 5B, indicating good separation near null-detuning (type 1 collision) while maintaining tight ZZ distribution with median 69 kHz (±23.2 kHz). We note that transmon-transmon coupling suffers from static-ZZ (i.e., “always-on”) error; however, within the Δ*f_j,k_* < δ regime (anharmonicity δ ≃ −330 MHz; see Materials and Methods), tailoring the ZZ distribution to present magnitudes (~70 kHz) with collision-free detuning is sufficient to yield gate errors approaching 10^−2^ for typical two-qubit CR gate durations of ~400 ns, indicating that our two-qubit gate fidelities are near levels set by coherence *(**22**)*.

Two-qubit gate errors as a function of qubit-pair detuning are displayed in Fig. 5C, with their associated distribution shown in the adjacent probability density map (right). Notably, our detuning distribution shows more gates with positive control-target detuning, as expected given the greater ZX interaction in the positive detuning region. A CR gate error model is also depicted in the figure (see Materials and Methods), where the shaded background (gray) indicates low-fidelity regions consistent with known frequency collisions described in (*23*). As part of our model, an optimizer routine incorporates a standard CR echo sequence with optional target rotary pulsing to determine usable two-qubit detuning regions within the straddling regime. For a gate error of 1%, a total usable frequency range of 380 MHz is available (optimized using *J =* 1.75 MHz in the depicted model), which reduces to 350 MHz (130 MHz) for error targets of 0.5% (0.1%). We note that our depicted model does not incorporate the impacts of classical cross-talk and coherence, the latter of which limits gate error in the desirable detuning regions. On the basis of the pre-LASIQ detuning distribution in Fig. 5A and the CR gate error model in Fig. 5C, we predict an initial mean gate error of 5.7%, which is improved to 1.4% after LASIQ tuning and demonstrates the efficacy of LASIQ in improving two-qubit gate fidelity (see the Supplementary Materials). Last, we note that although our collision bounds and unitary gate model have similar qualitative outcomes, further work is required to determine exact collision constraints and identify high-fidelity detuning regimes as lattice sizes are progressively increased.

## DISCUSSION

Significant yield improvement and high two-qubit gate fidelities for both Falcon and Hummingbird processors demonstrate LASIQ as an effective post-fabrication frequency trimming technique for multiqubit processors based on fixed-frequency transmon architectures. Selective laser annealing offers a compelling and scalable solution to the problem of frequency crowding, with LASIQ being readily adaptable to the scaling of qubits on progressively larger quantum processors. On the basis of Monte Carlo models (*23*), the 4.7-MHz frequency-equivalent resistance tuning precision of LASIQ allows high-yield scaling up to and beyond the 1000-qubit level. At present, cryogenic *f*_01_ measurements indicate a practical precision of 18.5 MHz; however, statistical analysis of cryogenic-to-ambient thermal cycling in a dilution refrigerator yields a recool stability of 5.7 MHz for our multiqubit processors, which may be leveraged to obtain *f*_01_(*R_n_*) predictions with similar accuracy and approach the baseline frequency assignment precision allowed by LASIQ. Last, we note that despite our present emphasis on NN collisions, errors arising from next-NN qubits (e.g., spectator collisions) are a rising concern with lattice scaling, and future work will incorporate tuning plans to minimize these errors, as well as lattice frequency pattern optimization for yield maximization.

## MATERIALS AND METHODS

### Laser annealing for transmon frequency allocation

The LASIQ frequency tuning system is depicted in Fig. 1A, whereby a diode-pumped solid-state laser (532 nm) is actively aligned and focused on a multiqubit quantum processor to selectively anneal individual transmon qubits (*23*). A timed shutter precisely controls the anneal duration and monotonically increments the junction resistance over multiple exposures, with intermediate resistance measurements to aid the adaptive approach to target resistances (*R_T_*). Diffractive beam shaping is optionally available to aid in the uniform thermal loading of the junction (*36*). The LASIQ process is fully automated and tunes an entire chip to completion, which occurs when each junction is annealed to within 0.3% tolerance around *R_T_* [corresponding to ~10 MHz for typical *f*_01_(*R_n_*) correlations].

### Preparation and screening of multiqubit processors

The Josephson junctions are fabricated via standard electron beam lithographic patterning followed by two-angle shadow evaporation with intermediate oxidation for Al/AlO_2_/Al junction formation (*37*). Typical lateral dimensions of the junction are ~100 nm, which yield as-fabricated frequency deviations near 2%, corresponding to ~100 MHz of qubit frequency spread. Transmon qubit designs follow those described in (*5*, *23*), with coupled qubits in a fixed-frequency lattice architecture. Nominal qubit anharmonicities are engineered near δ ≃ −330 MHz. Following junction evaporation, prescreening of viable processor candidates is performed via room temperature resistance measurements of all Josephson junctions, with transmon qubit frequencies estimated through wafer-level *f*_01_(*R_n_*) power-law fits from prior control experiments. Candidates are ranked on the basis of suitability for LASIQ tuning into collision-free tuning plans and within the Purcell filter bandwidth, all under the constraint of tuning range limits (typical maximum resistance tuning of ~14%). Following this preselection process, a frequency plan is generated and tested through Monte Carlo collision and yield modeling (*23*). Automated LASIQ tuning is performed as defined by the tuning plan for each transmon qubit to a junction resistance precision band of 0.3%, which yields an as-tuned RMS frequency-equivalent resistance precision of ~5 MHz (see the “LASIQ tuning precision”). After LASIQ, the multiqubit processors are plasma-cleaned and flip-chip bonded to an interposer layer for Purcell filtering and readout. The bonded processors are mounted into a dilution refrigerator for cryogenic screening, including coherence measurements and single- and two-qubit gate error analysis. The post-LASIQ cleaning, bonding, and mounting steps are coordinated to occur within a 24-hour span to minimize junction aging and drifts, thereby maintaining the qubits faithfully outside collision bounds of the intended tuning frequency plans.

### Gate error model

Our gate error estimates are the result of time-domain simulations of the Schrodinger equation within the Duffing model of two coupled transmon qubits (*33*). CR pulses are modeled as “Gaussian square” in shape. For each set of parameters, CR gate amplitudes are tuned up in a two-pulse echo for a fixed gate time by minimizing the Bloch vector (*5*). A rotary pulse is additionally applied to the target qubit during the CR pulses, the amplitude of which is optimized to minimize gate error, as described in (*22*). Using an adaptive Runge-Kutta solver, we calculate the full unitary evolution of the system resulting from the calibrated gate sequence. From this simulated unitary U, error can be estimated by *E =* 1 –[1/*d × |*Tr(U_target_U^†^)*|*^2^ *+* 1]/(*d +* 1) (where *d =* 2*^n^* and *n =* 2 for our two-qubit simulations) and is compared to the results of two-qubit randomized benchmarking.
