# Supplementary material for: High-performance superconducting quantum processors via laser annealing of transmon qubits
Source: Sci Adv. 2022 May 13;8(19):eabi6690. doi: 10.1126/sciadv.abi6690 (PMC9106287; doi:10.1126/sciadv.abi6690)
Supplement: Supplementary file 1 — Supplementary Text Figs. S1 to S4 [file sciadv.abi6690_sm.pdf]

Science Advances
AAAS

Supplementary Materials for

High-performance superconducting quantum processors via laser annealing of transmon qubits

Eric J. Zhang*, Srikanth Srinivasan, Neereja Sundaresan, Daniela F. Bogorin, Yves Martin, Jared B. Hertzberg, John Timmerwilke, Emily J. Pritchett, Jeng-Bang Yau, Cindy Wang, William Landers, Eric P. Lewandowski, Adinath Narasgond, Sami Rosenblatt, George A. Keefe, Isaac Lauer, Mary Beth Rothwell, Douglas T. McClure, Oliver E. Dial, Jason S. Orcutt, Markus Brink, Jerry M. Chow

*Corresponding author.email: eric.jh.zhang@ibm.com

Published 13 May 2022, *Sci. Adv.* **8**, eabi6690 (2022)
DOI: 10.1126/sciadv.abi6690

This PDF file includes:

Supplementary Text
Figs. S1 to S4

Supplementary Text

In this supplementary section we present and expand upon results of (A) Monte Carlo yield modeling of the tuned (and comparison against the untuned) 27-qubit *Falcon* processor presented in Sec II-A, and (B) Statistical analysis of tuning success rates as described in Sec. II-B.

A. 27-qubit *Falcon* yield

In Sec II-A, an example 27-qubit *Falcon* processor was LASIQ-tuned for nearest-neighbor (NN) type 1-4 collision removal. To ascertain the impact of tuning on the NN collision-free yield rates, a Monte Carlo simulation *(23)* has been performed for the initial (pre-tuned) and final (post-tuned) transmon qubit frequencies, as predicted by typical power-law $f_{01}(R_n)$ curves for our sample. As described in *(23)*, our model incorporates a random deviation (i.e. normal spread) from the target qubit frequencies, which may arise from empirical qubit spread from cleaning, bonding and cooldown processes (18.5 MHz), tuning imprecision (4.7 MHz), or other factors resulting in deviations from the desired target (Sec. II-B). We model the yield by adding to each target qubit frequency a random variation consistent with this frequency spread and count the proportion of NN collision-free outcomes.

Fig. S1 shows the result of our yield models, indicating both the untuned (red) and tuned yield rates (blue) as a function of target frequency deviation. As-fabricated, the chip yields a significant number of type 1-4 NN collisions, as evidenced by the poorly conditioned untuned yield curve (red). The low yields for small σ_f results from the presence of existing collisions immediately after fabrication and prior to tuning. However, even accounting for large random spreads, the initial untuned lattice frequency pattern is highly collision prone, with yields remaining under 5% for σ_f < 40 MHz. The poorly conditioned untuned yield curve is a general characteristic of fixed-frequency architectures that exhibit frequency crowding. The primary goal of our LASIQ tuning method to arrange our lattice frequency into well-conditioned yield curves with high probability of achieving a NN collision-free configuration.

Fig. S2 tabulates the various NN collision types, as well as tolerance bounds around which collision avoidance is successfully achieved. As described in the main text and elaborated in *(23)*, four main collision types are enumerated. Type-1 corresponds to two-qubit hybridization, with degenerate $|0\rangle \rightarrow |1\rangle$ control (*j*) and target (*k*) level spacings. Type-2 results from two-photon excitation of *j* into the non-computational $|2\rangle$ state, and type-3 poses similar risk when $f_{j,01} = f_{k,12}$ and vice-versa. Type-4 corresponds to a 'slow gate,' whereby reduced ZX interaction strength occurs due to two-qubit detuning beyond the straddling regime, resulting in longer gate operating times. The Δ_c collision bounds are displayed in the third column of Fig. S2, numerically determined to yield ≤ 1% two-qubit gate errors *(23, 33)*, and are consequently utilized in the Monte Carlo collision and yield models depicted in Fig. S1. In the process of generating tuning plans for LASIQ, we generally adopt the 'best practice' protocol of doubling these to $2\Delta_c$ bounds shown in the final column of Fig. S2, which provide tuning plans with greater robustness to post-tuned drifts, and therefore yields NN collision-free tuned candidates with increased confidence.

The tuning plan for this particular *Falcon* processor is outlined in Fig. 1(a), with the aim of tuning out NN collisions and ensuring frequencies reside within the Purcell filter bandwidth, while maintaining resistance tuning < 14%. All qubits are successfully tuned to frequency targets with a predicted 4.8 MHz precision, as outlined in Sec II-A. The resulting Monte Carlo yield model of the tuned lattice pattern is shown by the well-conditioned (blue) curve in Fig. S1, indicating a typical yield roll-off as the frequency deviation increases. For nominal practical spreads (~20 MHz, Sec. II-B), we calculate a yield improvement of 15×, from 3.4% to 51%, indicating the efficacy of the LASIQ tuning method. A similar comparison is performed for the baseline precision achievable by LASIQ, which is predicted at 4.8 MHz for this chip (based on 0.16% post-LASIQ resistance precision) and indicates NN collision-free yields beyond 90%. As lattice sizes are scaled to 10^3 qubit levels, practical tuning precisions approaching these single-MHz levels will be required to ensure appreciable yields. Presently, a major contributor to our practical trimming precision results from the imprecision of predicting f_{01} from R_n *(23)*, and significant gains may be achieved by leveraging processor recool cycles to tailor $f_{01}(R_n)$ predictions. In particular, statistics on qubit frequency deviations during cryogenic cycling yields a recool stability of 5.7 MHz (see Sec. III), which provides a practical pathway to approach the baseline precision of ~5 MHz set by the frequency-equivalent resistance precision of our LASIQ process.

B. Tuning success statistics

In Sec. II-B, a tuning sample of 349 *Falcon* qubits out of 390 total qubits were tuned to resistance targets (R_T), spanning a tuning range up to 14.5% with 89.5% success rate. In this section we break down the tuning success statistics of the tuned qubits, and outline failure mechanisms which induce deviations from target resistances extending beyond the desired target band.

Fig. S3 shows the tuning statistics of all 390 qubits used in our precision benchmarking experiments. The bottom panel indicates the desired tuning histogram distribution (red, striped) with respect to initial junction resistance, superimposed upon the successfully tuned qubits (gray). Tuning success is defined as tuning to within 0.3% band around R_T, and the LASIQ system will proceed incrementally towards this goal. A Gaussian distribution of the successfully tuned qubits is also depicted, with a mean tuned distance of 7.2% (σ = 3.5%). We note here that each of the *Falcon* qubits was tuned as part of a tuning plan designed for NN collision avoidance, and hence our tuning distances are representative of realistic processor samples. It is notable that deviations between the desired (red, striped) and actual (gray) tuning distributions increasingly depart at both lower and higher tuning ranges, indicating that missed targets occur at the tuning distribution extremities.

The central panel of Fig. S3 indicates the cumulative plot of LASIQ tuned qubits, with both tuned total (red) and successfully tuned (black) qubits shown. Corresponding success rates for each tuning distance (assessed at 1% intervals) are displayed in the top panel, which indicates that failure rates are most likely (< 90% success) for tuning targets under 1% and above 10%. Below, we consider each case in turn.

In the former case, small desired tuning distances are exposed to risk of overshooting (i.e. $\Delta R > R_T - R_n$). This is a result of the statistical spread in resistance progression, and small desired resistance shifts suffer increased risk of accidental increase beyond desired targets. Despite this

risk, we note that typical optimal frequency separation between qubits in our tuning plans are on the order ~100 MHz, and even for significant overshoot, NN collisions are unlikely to be induced unless the deviation between tuned resistances and targets approach the frequency spacing between NN qubits.

In the latter case, undershoot effects appear for junction targets beyond $\Delta R > 10\%$, as evidenced by the slow tapering down of success rates in the top panel of Fig. S3. This is a consequence of junctions reaching their maximum tuning limit, which based on calibration measurements on junction arrays, is near 14%, with a spread of $\sigma \sim 2\%$. In almost all cases, tuning plans are engineered to reside under 14% tuning, to ensure that almost all qubits on a given processor have high probability of reaching desired targets. Nevertheless, the statistical nature of the tuning range causes outlier qubits which saturate at lower ΔR, resulting in monotonically decreasing success rates as observed in Fig. S3. Progress in scaling lattices of transmon qubits will benefit greatly from determining the origin of this resistance limit, and the best-practice fabrication processes through which this may be increased.

C. Comparing pre- and post-LASIQ gate error rates

Sec. II-C describes an operational LASIQ-tuned 65-qubit *Hummingbird* processor, along with the characterization of two-qubit gate-errors. The role of LASIQ tuning is primarily to engineer the two-qubit detuning distribution to reside outside of collision boundaries (enumerated and described in Fig. S2) and within the straddling regime for high-ZX interaction *(33)*. Our *Hummingbird* processors have a total of 72 two-qubit gates, and the tuning plans are engineered to ensure all gates are operational by reducing the errors of those two-qubit pairs with sub-optimal detuning. The pre- and post-LASIQ tuned two-qubit detuning distributions are shown in Fig. 5(a), while Figs. 5(b) and 5(c) respectively show the final engineered ZZ distribution and measured two-qubit gate error rates. Our interposer bonding process for our *Hummingbird* processors precludes the ability to perform a direct pre- and post-LASIQ measurement of gate-errors; however, we present below a comparison based on predicted initial ZZ and two-qubit gate-error rates to demonstrate the efficacy of LASIQ tuning in scaling our multi-qubit processors.

Fig. S4 shows a comparison before and after LASIQ tuning of our 65-qubit *ibmq_manhattan* processor. Fig. S4(a) depicts a comparison of the ZZ statistics, where the blue points are the untuned two-qubit gate pairs prior to LASIQ, as calculated from the extracted J coupling, initial two-qubit detuning and anharmonicity $\delta \simeq -330$ MHz of the qubits *(33)*. The orange points are the measured ZZ after LASIQ tuning, which is also presented in Fig. 5(b). Notably, the pre-LASIQ ZZ distribution includes a significant number of two-qubit pairs residing within collision zones, and particularly several outliers with predicted initial ZZ well above 100 kHz. After LASIQ tuning, the distribution (orange) is seen to have been shifted outside of the collision zones, and the high-ZZ outliers have been successfully eliminated.

The bottom panel, Fig. S4(b) compares the two-qubit gate error rates (ε) before (blue) and after (orange) LASIQ tuning. We note that all 72 two-qubit gates are measured on our *ibmq_manhattan* processor, giving a 100% yield of working two-qubit gates. The gray dashed line is a heuristic determination of two-qubit errors set by the limits of coherence *(22)*, given our measured T_1 and T_2 (Fig. 4) and typical gate times ~400 ns. This coherence-limited gate-error rate ~0.9% is

consistent with the measured values of the post-tuned two-qubit gates outside of the known collision zones. To predict the pre-tuned gate error rates, we use the initial two-qubit detuning and our CR gate error model (see Sec. II-C), which shows a significant number of gates residing within low-fidelity collision regions. We note that in Fig. S4(b), only those two-qubit gates with predicted error above the coherence limit are shown; gates where the CR gate error model predicts error rates below the coherence limit (gray line) are assumed to be limited at ~0.9%. Comparing the mean gate error rates (ε) of the pre- and post-LASIQ tuned two-qubit gates, we find that gate errors decrease from $\langle \varepsilon_{untuned} \rangle$ = 5.7% to $\langle \varepsilon_{tuned} \rangle$ = 1.4% and are no longer limited by frequency collisions. We anticipate that further improvements in coherence and other functional parameters of our multi-qubit systems will drive this limit down and approach the limits required for fault-tolerant computing.

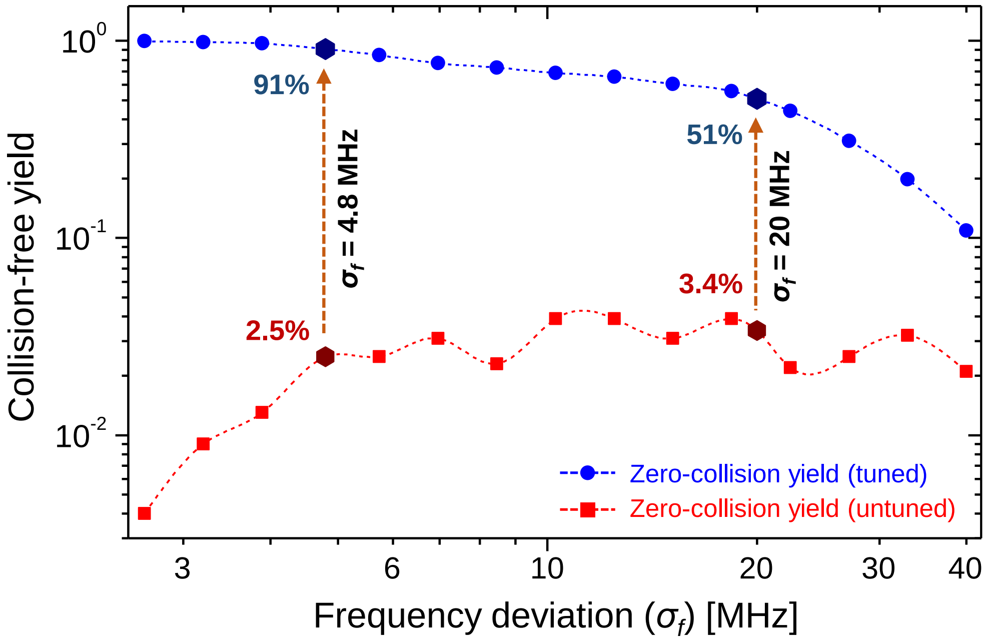

Fig. S1.

Monte Carlo yield modeling for nearest-neighbor (NN) collision-free operation on a 27-qubit *Falcon* processor. Zero-collision yield modeling is based on pre- and post-LASIQ frequency predictions determined in Sec II-A (Fig. 1). Pre-tuning (red), the yield curve is poorly conditioned and indicates < 5% NN collision-free yield for frequency deviations up to 40 MHz. Low yields for small spreads are due to the presence of initial type 1-4 collisions that exist on the as-fabricated chip. Post-tuning, the final yield curve (blue) indicates ≥ 10× improved yield rates. For nominal post-tuned spreading (~20 MHz, Sec. II-C), our models indicate 51% NN collision free yield. At the limit of LASIQ tuning precision predicted for this chip (4.8 MHz), this yield improves to above 90%.

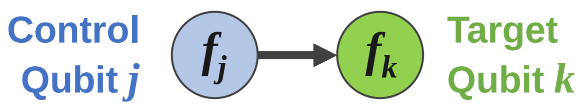

Type	Condition	Bounds (Δ_C)	Bounds ($2\Delta_C$)
1	$\lvert\Delta f_{j,k}\rvert = 0$	± 17 MHz	± 34 MHz
2	$\lvert\Delta f_{j,k}\rvert = \delta/2$	± 4 MHz	± 8 MHz
3	$\lvert\Delta f_{j,k}\rvert = \delta$	± 30 MHz	± 60 MHz
4	$\lvert\Delta f_{j,k}\rvert > \delta$	(slow gate)	(slow gate)

Fig. S2.

Nearest-neighbor (NN) frequency collision bounds. The bounds depicted above are utilized in Monte Carlo yield modeling and the generation of NN collision-free tuning plans. Four collision types are identified, which correspond to level hybridization (type-1), excitation of the control or target qubit into the non-computational $|2\rangle$ state (type-2 and type-3), as well as 'slow gates' (type-4) where excess two-qubit detuning results in diminished ZX interaction. Qubit anharmonicities are engineered near $\delta \simeq -330$ MHz (see Materials and Methods). The quantitative collision bounds (Δ_c) corresponding to gate errors below 1% are displayed in the third column, as determined in *(23, 33)*. The final column ($2\Delta_c$) indicates double bounds utilized in generating NN collision-free tuning plans, serving as a 'best practice' protocol for successfully yielding NN collision-free candidates.

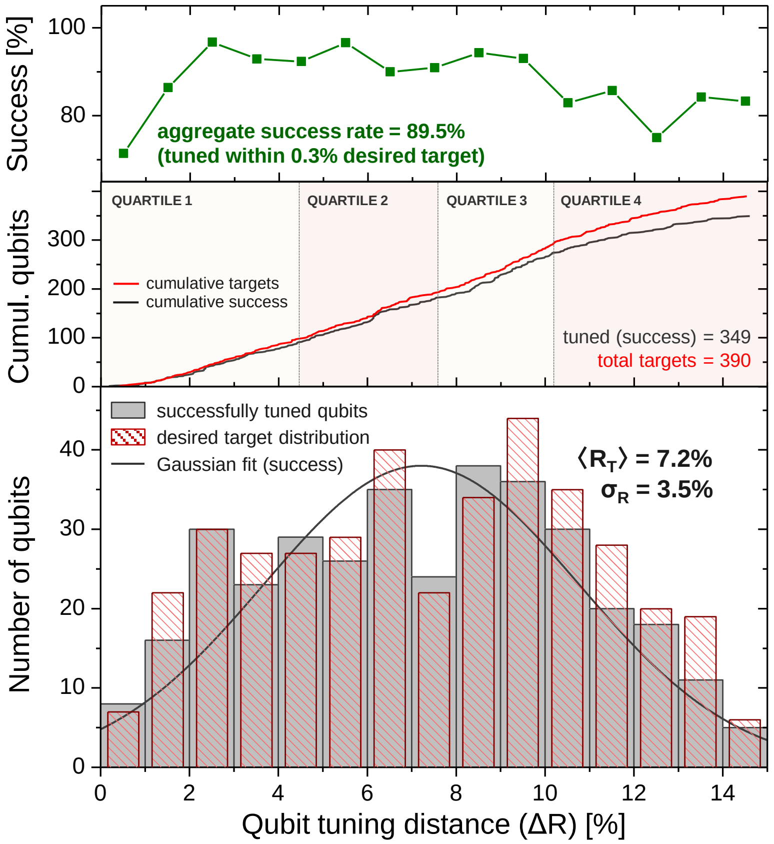

Fig. S3.

Aggregate success statistics of 390 qubits from trial tuning iterations of *Falcon* processors. The bottom panel indicates the desired tuning resistance targets and actual tuning distance (ΔR, shown by the striped red and shaded gray histograms respectively). A Gaussian distribution over the successfully tuned qubits (349 out of 390) is shown, with a mean tuning range of 7.2 ± 3.5%. The cumulative number of tuned qubits is shown in the middle panel, with total tuned qubits (red) and successfully tuned qubits (black) displayed for comparison. The deviations demonstrate that at higher desired tuning distances, a greater failure rate occurs due to difficulty reaching the desired ΔR. This is further corroborated in the top panel (green squares) indicating the tuning success rate for each ΔR class of qubits. Near the wings of the curve, we observe dips in success rate, with the junctions with lower target ΔR suffering generally from overshoot, whereas higher target ΔR values suffer from undershoot. The total aggregate success rate (defined as tuning to within 0.3% target resistance band) is 89.5%.

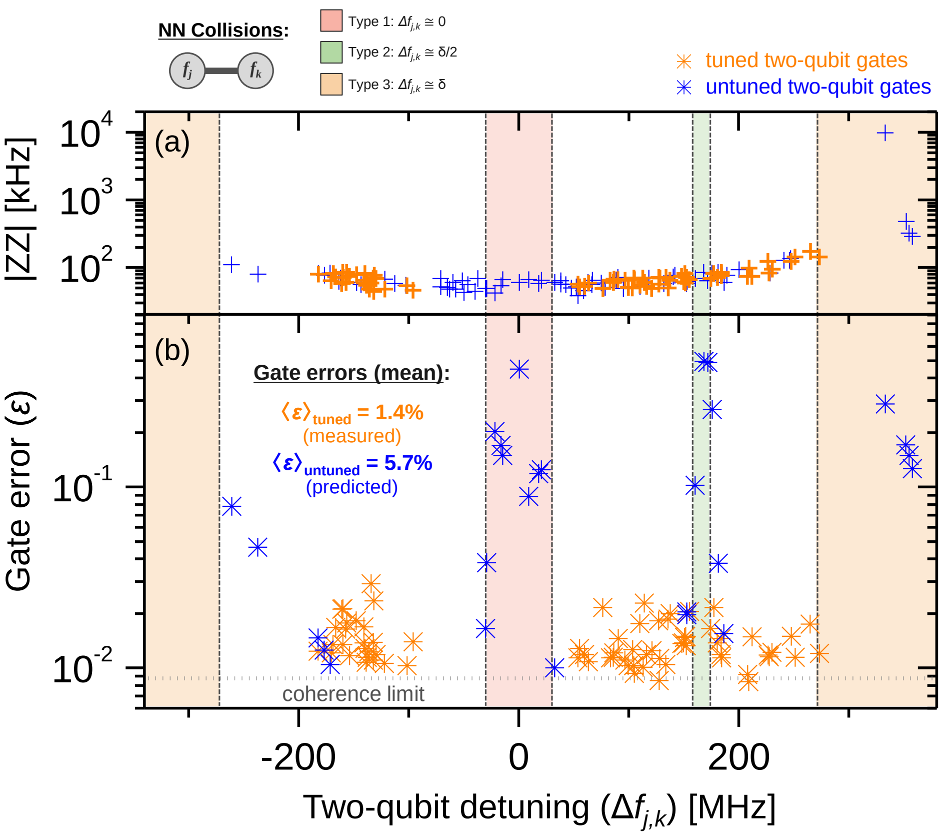

Fig. S4.

Comparison of pre-tuned (predicted) and post-tuned (measured) two-qubit gate errors. (a) Initial pre-tuned ZZ values (blue) are predicted based on the initial two-qubit detuning, the extracted J coupling for each two-qubit pair and the measured anharmonicity ($\langle\delta\rangle = -332$ MHz, Fig. 5(b)) *(33)*. The goal of LASIQ tuning is to engineer the ZZ distribution away from collision zones (shaded regions) whilst maintaining low ZZ. The post-tuned measured ZZ values are overlayed in orange and yields a median of 69 kHz with a spread of 23.2 kHz. (b) Predicted two-qubit gate errors (blue) based on our CR gate error modeling *(35)* from Fig. 5(c) of the main text. The gray dashed line is a heuristic calculated from typical gate times (~400 ns) and coherence times (Fig. 4). For clarity, only those initial two-qubit pairs with error rates above the limit set by coherence are depicted. A mean error rate of 5.7% is predicted for the 72 untuned two-qubit gates, whilst after LASIQ tuning, the mean error rate is improved to 1.4% (orange).
